# The western Mediterranean region provided the founder population of domesticated narrow-leafed lupin

**DOI:** 10.1007/s00122-018-3171-x

**Published:** 2018-09-17

**Authors:** Mahsa Mousavi-Derazmahalleh, Bruno Nevado, Philipp E. Bayer, Dmitry A. Filatov, James K. Hane, David Edwards, William Erskine, Matthew N. Nelson

**Affiliations:** 1 UWA School of Agriculture and Environment, The University of Western Australia, 35 Stirling Highway, Crawley, WA 6009 Australia; 2 Department of Plant Sciences, University of Oxford, Oxford, OX1 3RB UK; 3 School of Biological Sciences, The University of Western Australia, 35 Stirling Highway, Crawley, WA 6009 Australia; 4 CCDM Bioinformatics, Centre for Crop and Disease Management, Curtin University, Bentley, WA 6102 Australia; 5 The UWA Institute of Agriculture, The University of Western Australia, 35 Stirling Highway, Perth, WA 6009 Australia; 6 Centre for Plant Genetics and Breeding, UWA School of Agriculture and Environment, The University of Western Australia, 35 Stirling Highway, Crawley, WA 6009 Australia; 7 Natural Capital and Plant Health, Royal Botanic Gardens Kew, Wakehurst Place, Ardingly, West Sussex RH17 6TN UK

## Abstract

**Key message:**

This study revealed that the western Mediterranean provided the founder population for domesticated narrow-leafed lupin and that genetic diversity decreased significantly during narrow-leafed lupin domestication.

**Abstract:**

The evolutionary history of plants during domestication profoundly shaped the genome structure and genetic diversity of today’s crops. Advances in next-generation sequencing technologies allow unprecedented opportunities to understand genome evolution in minor crops, which constitute the majority of plant domestications. A diverse set of 231 wild and domesticated narrow-leafed lupin (*Lupinus angustifolius* L.) accessions were subjected to genotyping-by-sequencing using diversity arrays technology. Phylogenetic, genome-wide divergence and linkage disequilibrium analyses were applied to identify the founder population of domesticated narrow-leafed lupin and the genome-wide effect of domestication on its genome. We found wild western Mediterranean population as the founder of domesticated narrow-leafed lupin. Domestication was associated with an almost threefold reduction in genome diversity in domesticated accessions compared to their wild relatives. Selective sweep analysis identified no significant footprints of selection around domestication loci. A genome-wide association study identified single nucleotide polymorphism markers associated with pod dehiscence. This new understanding of the genomic consequences of narrow-leafed lupin domestication along with molecular marker tools developed here will assist plant breeders more effectively access wild genetic diversity for crop improvement.

**Electronic supplementary material:**

The online version of this article (10.1007/s00122-018-3171-x) contains supplementary material, which is available to authorized users.

## Introduction

The ancestral origin of crop species is one of the primary questions in plant domestication. Current distribution of wild progenitors of crops often provides a reliable indicator of where domestication occurred. While the answer to this question is well known for some crops such as wheat (*Triticum aestivum*) and lentil (*Lens culinaris*) (Erskine 1998; Heun et al. 1997), our understanding of other crops remains limited (Zohary et al. 2012). Unravelling fundamental stages in the evolution of domesticated plants is another keystone of domestication research. This includes isolating and characterising domestication genes, inferring historic population bottlenecks and gene flow based on extant patterns of genetic diversity and the accumulation of yield- and quality-related minor genes. The emergence of new genomic tools has both revolutionised the precision of these studies and extended their breadth beyond the major staple crop species (Emshwiller 2006; Gepts 2014; Larson et al. 2014; Mousavi-Derazmahalleh et al. 2018a).

Plant domestication involved the incorporation of traits that made them more amenable to agriculture. Repeatedly, certain traits were fixed in a range of grain crop species, which are collectively known as domestication syndrome traits (Hammer 1984; Zohary et al. 2012). These traits include enlarged fruit or grain, changes in photoperiod or vernalisation sensitivity, reduced seed dehiscence and reduced anti-nutritional content. Genes and mechanisms underlying those phenotypic changes have been identified through different methods, which can be thought of as ‘top-down’ (quantitative trait locus (QTL) and linkage disequilibrium (LD) mapping studies) and ‘bottom-up’ (demographic approach and empirical ranking) approaches as described by Ross-Ibarra et al. (2007). QTL mapping studies using biparental populations have successfully identified the genomic regions associated with the domestication syndrome in crops such as wheat (Peng et al. 2003), rice (Xiong et al. 1999), tomato (Frary et al. 2000), common bean (*Phaseolus vulgaris* L.) (Koinange et al. 1996) and adzuki bean (*Vigna angularis*) (Isemura et al. 2007). More recently, LD mapping using diverse sets of accessions has become more prominent including genome-wide association studies (GWAS) in sorghum to identify inflorescence architecture genes and plant height loci (Morris et al. 2013), identifying candidate genes for starch content regulation in maize kernel (Liu et al. 2016), and detecting genes related to agronomic traits in rice (Yano et al. 2016) and soybean (Zhang et al. 2015). ‘Bottom-up’ demographic-based approaches have been less widely applied in plant diversity studies, with successful examples provided in maize (Wright et al. 2005) and *Arabidopsis halleri* (Fischer et al. 2013) where regions of genome associated with selection and adaptation were successfully identified.

Advances in genomic technology now make it practical to extend our horizons beyond the main staple crops. The genus *Lupinus* offers a number of features that make it amenable to domestication analysis. The genus includes four species that were independently domesticated across contrasting eras and locations (Wolko et al. 2011). *Lupinus* is a diverse genus of c. 280 annual and perennial species within the genistoid clade of legumes (Eastwood et al. 2008). The rate of species diversification within *Lupinus* is the highest of any known plant genus and so presents a useful model to understand evolution (Hughes and Eastwood 2006). While most lupin grain is used for livestock feed, there is increasing interest in using lupin grain as a health food for humans because it is gluten-free, high in protein and dietary fibre, low in fat and starch content and demonstrates anti-diabetes and anti-inflammatory properties (Foley et al. 2011; Lima-Cabello et al. 2018). Furthermore, the content of anti-nutritive factors (inhibitors of proteinase and hemagglutinins) in lupin seed protein is lower than other legumes which adds to the value of its seed for consumption (Kurlovich et al. 2003). Finally, the high quantity and quality of protein in lupin seed make it suitable for aquaculture feed (Robaina et al. 1995). Of the four widely cultivated species of *Lupinus,* narrow-leafed lupin (*L. angustifolius*), white lupin (*L. albus*) and yellow lupin (*L. luteus*) are from the Old World (Mediterranean region/North and East Africa), whereas Andean lupin or tarwii (*L. mutabilis*) is from the New World (North/South America) (Wolko et al. 2011).

Narrow-leafed lupin is unique among crops in that its domestication is fully documented within the scientific literature of the twentieth century (Gladstones 1970). Its modern breeding started with the discovery by von Sengbusch in Germany in 1928/1929 of the recessive mutant allele *iucundus*, which controls alkaloid production. Over the next 30 years, breeders identified and incorporated further domestication genes: removal of physical seed dormancy (*mollis)*, two genes responsible for reduced pod shattering (*tardus* and *lentus*), changed flower and seed colour as a marker of domestication (*leucospermus*) and an early flowering gene which removed the vernalisation requirement (*Ku*) (Cowling et al. 1998; Nelson et al. 2006).

Narrow-leafed lupin domestication took place within a short time frame and involved a series of severe genetic bottlenecks (Berger et al. 2012). The short segmented domestication history of the crop indicated that only a small proportion of its genetic and adaptive diversity potential was incorporated into the domesticated gene pool. Thus, a major breeding priority for this species is to transfer genetic and adaptive diversity from wild germplasm into the domesticated gene pool (Berger et al. 2013). A recent study of the three Old World lupin species (*L. angustifolius*, *L. albus* and *L. luteus*) demonstrated that phenology in all three species has been under strong selection along aridity gradients (Berger et al. 2017). Furthermore, a recent study of New World lupin species showed there is more frequent genome-wide adaptation in rapidly diversifying species, as opposed to slowly diversifying species and plant species more generally (Nevado et al. 2016). Additionally, while early phenotypic studies suggested the Aegean region as the diversity centre of wild narrow-leafed lupin (Clements and Cowling 1994), we recently showed that the western Mediterranean is the centre of genetic diversity. We also demonstrated a strong east–west differentiation among wild narrow-leafed lupin across Mediterranean basin and historic migration from west to east (Mousavi-Derazmahalleh et al. 2018b).

The recent publication of a comprehensive reference genome for narrow-leafed lupin (Hane et al. 2017) provides an excellent opportunity to use this species as a model to characterise the evolutionary selection that accompanies plant domestication, in order to address the following questions: (I) What is the genome-wide impact of domestication? (II) What is the founder population of domesticated narrow-leafed lupin? (III) Are footprints of selection evident at domestication loci?

## Materials and methods

### Plant materials

A total of 146 wild and 85 domesticated accessions of narrow-leafed lupin, representative of a wide range of genetic diversity, were obtained from the Australian Lupin Collection, Department of Agriculture and Food Western Australia (DAFWA; Online Resource 1). Details of wild accessions were described by Mousavi-Derazmahalleh et al. (2018b). Domesticated accessions are from seven countries (Australia, Belarus, Germany, Poland, Russia, Ukraine and South Africa), which cover all the major breeding programmes of narrow-leafed lupin. Information on country of origin, wild/domestic status and phenotypes of these accessions is provided in Online Resource 1. Three plants per domesticated accession were grown in a Phytotron with approximate day lengths of 13–14 h and average temperature of 20 °C. Alkaloid status and flower colour were assessed on the domesticated accessions, and leaf samples were taken a single representative plant from each accession for DNA extraction (Online Resource 1). These phenotypic data were supplemented by previously published data on alkaloid status, pod dehiscence, physical dormancy (hard versus soft seededness), rain, soil pH at collection site, flower colour, time to flowering from sowing date (flowering time), height at maturity and 100-seed weight (Gladstones and Crosbie 1979; Online Resource 1). Seven accessions (P26107, P22845, P22839, P26446, P26562, P27913 and P28485) had ambiguous seed water permeability status and so were treated as missing values (NA) to avoid potential bias in downstream analysis.

### DArTseq genotyping

 We extracted DNA from leaves of single plants from each accession using a modified CTAB method (Doyle and Doyle 1990). The quality and quantity of extracted DNA were assessed using standard agarose electrophoresis and Qubit assays (www.Invitrogen.com/qubit). The DNA concentration of each sample was adjusted to 20 ng/μL and subjected to DArTSeq™ (hereafter, DArTseq) genotyping at Diversity Arrays Technology Pty Ltd, Canberra, Australia (Sansaloni et al. 2011). Technical replicates (same DNA extraction) were also included for reference accessions P27255 and 83A:476. The sequence data were processed by the DArT P/L in-house analytical pipeline.

Bi-allelic SNP markers were identified for downstream analyses and were filtered based on different thresholds for different analyses. SNP markers, which had positions mapped to pseudo-chromosomes in the *L. angustifolius* cv. Tanjil reference genome (Hane et al. 2017), were used for Fst and GWAS analyses. For phylogeny, population structure and linkage disequilibrium analyses, SNPs with more than 25% missing data or 12.8% heterozygosity were eliminated. DArTseq reads were aligned with the Tanjil reference genome using the nucmer aligner (Delcher et al. 1999), setting the minimum cluster length (-c) to 25 bp and ≤ 3 matches. The latter threshold was selected to take account of the remnants of whole-genome triplication in the genome of narrow-leafed lupin (Hane et al. 2017; Kroc et al. 2014). Then, the positions of SNPs relative to the Tanjil reference genome were determined from SNP positions on the DArTseq reads and the reads’ match position on the reference, summarised in variant call format (VCF) relative to the lupin reference genome (Hane et al. 2017). The VCF file was validated using the Genome Analysis Toolkit (GATK) (McKenna et al. 2010).

### Assessing phylogenetic relationship, structure and genetic diversity within germplasm

To infer the likely origin of domesticated samples, we defined three groups (western, eastern and central Mediterranean) within the wild narrow-leafed lupin with respect to samples’ geographical boundaries (Online Resource 1). We then estimated the genome-wide divergence between each of these wild populations and the domesticated narrow-leafed lupin samples, using windows of size 1 Mb in mstatspop (available from https://bioinformatics.cragenomica.es/numgenomics/people/sebas/software/software.html). This method uses the Dxy measure, which is the average number of pairwise differences between each individual of one population and each individual of other population (Cruickshank and Hahn 2014; Nei 1987).

MrBayes v3.2.2 (Ronquist et al. 2012), Bayesian inference of phylogeny estimating the posterior probability distribution of all possible phylogenies under Markov chain Monte Carlo (MCMC), was conducted to analyse the phylogeny. Four chains of MCMC, assuming general time reversible (GTR) model of molecular evolution with gamma-distributed rate variation across sites, was run for 1,000,000 generations and sampled every 1000 generations. Then, the cladogram tree was plotted using Fig Tree v1.4.2 (http://tree.bio.ed.ac.uk/software/figtree/).

Two different methods were employed to identify population structure. The principal component analysis (PCA) was performed using EIGENSTRAT to assess genetic diversity and to correct for population stratification (Price et al. 2006). We also used fastSTRUCTURE for population numbers ranging from *K* = 2 to *K* = 12, using default parameters (Raj et al. 2014). The estimation of optimum *K* was obtained using the algorithm implemented in fastSTRUCTURE to choose model complexity (Raj et al. 2014).

Linkage disequilibrium (LD) as measured by *r*^2^ was calculated for all values (--ld-window-r2 0) for every SNP within a window of 1 Mb using Plink (Purcell et al. 2007). The mean *r*^2^ values pooled over all 20 chromosomes for domesticated (European and Australian) and wild germplasm were calculated and plotted using R v3.3.0 (R Core Team 2016). Average nucleotide diversity was measured as pi on a per-site basis using VCFtools (Danecek et al. 2011).

A genome-wide association study (GWAS) of SNPs with traits of interest was investigated using GAPIT (Lipka et al. 2012) with the first two principal components as covariates (this was applied as an option within the GAPIT programs and corrects for the population stratification) for an additive model and a minor allele frequency (MAF) cut-off of 0.01.

### Selective sweep analyses

A selective sweep affecting the domesticated samples after split from wild accessions would be expected to show high Fst (between wild and domesticated) and low variability of domesticated relative to wild accessions. To infer whether the candidate genes exhibited the expected signatures of selection, we performed a genome-wide scan using overlapping windows of size 200 kb and steps 50 kb. For each window, we used mstatspop to estimate Fst between domesticated and wild accessions and variability (theta and pi) within wild and domesticated accessions separately. To take into account the levels of ancestral variability within wild narrow-leafed lupin, we estimated the amount of polymorphism lost since domestication by calculating the relative amount of polymorphism within domesticated compared to wild, i.e. polymorphism (domesticated)/polymorphism (wild). In particular, we scanned domestication loci using coordinates provided by Hane et al. (2017) for evidence of selective sweeps. In order to take into account the false discovery rate (FDR), we used BayeScan 2.0 with FDR of 0.01 and 0.05 (Foll and Gaggiotti 2008), to identify Fst outliers stringently.

## Results

Phenotyping of 85 domesticated accessions supported previous publications (Gladstones and Crosbie 1979) and information provided by Australian Lupin Collection (Online Resource 1) and enabled these independent datasets to be merged. DArTseq analysis generated 45,230 co-dominant SNP markers in 146 wild and 85 domesticated narrow-leafed lupin accessions. For the Fst and GWAS analyses, we used a subset of 38,948 SNP markers that was mapped to pseudo-chromosomes and 11,690 SNPs were utilised for phylogeny, population structure and linkage disequilibrium studies. The allelic profile per accession for these 45,230 and 11,690 SNP sets are reported in the Online Resources 2 and 3, respectively.

### Identifying the founder population of domesticated narrow-leafed lupin

**Fig. 1:**
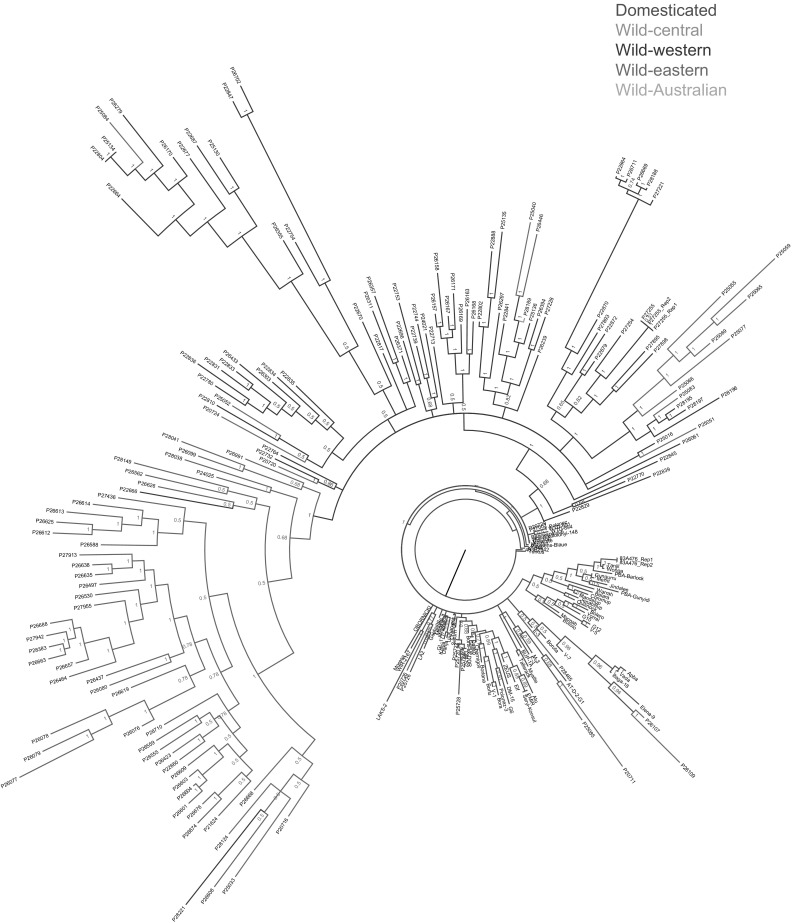
Phylogenetic tree of wild and domesticated narrow-leafed lupin constructed from 11,690 SNPs, using MrBayes v3.2.2. Accessions are colour coded based on their status (wild central Mediterranean [green; 21 accessions], wild western Mediterranean [navy blue; 77], wild eastern Mediterranean [purple; 49 accessions], wild Australian [pink; 1 accession], domesticated [red; 87 accessions])

Phylogenetic analysis clearly distinguished wild from domesticated accessions, and four wild accessions from the western Mediterranean (P22839 and P22829 from Portugal, P22770 from Spain and P22845 from Morocco) were basal to the domesticated accessions (Fig. 1). An unrooted radial tree version of the same figure is available as Online Resource 4. A NeighborNet network analysis showed similar pattern to the phylogeny results (Online Resource 5). This result was complimented by analysis of 478 1 Mb windows using mstatspop approach, which revealed the closest wild population to the domesticated accessions was the west population (*n* = 212), followed by the central population (*n* = 196), and lastly the east population (*n* = 70). Taken together, these findings rule out the eastern and central wild populations as the origin of domesticated narrow-leafed lupin and point to a western Mediterranean origin. However, within the cluster of domesticated accessions were two accessions from the eastern Mediterranean (P25085 and P28485) and four accessions from the central Mediterranean (P26107, P26109, P20711 and P20726) (Fig. 1). Technical replicates of reference accessions P27255 and 83A:476 showed high reproducibility of genotyping results (Fig. 1).

**Fig. 2:**
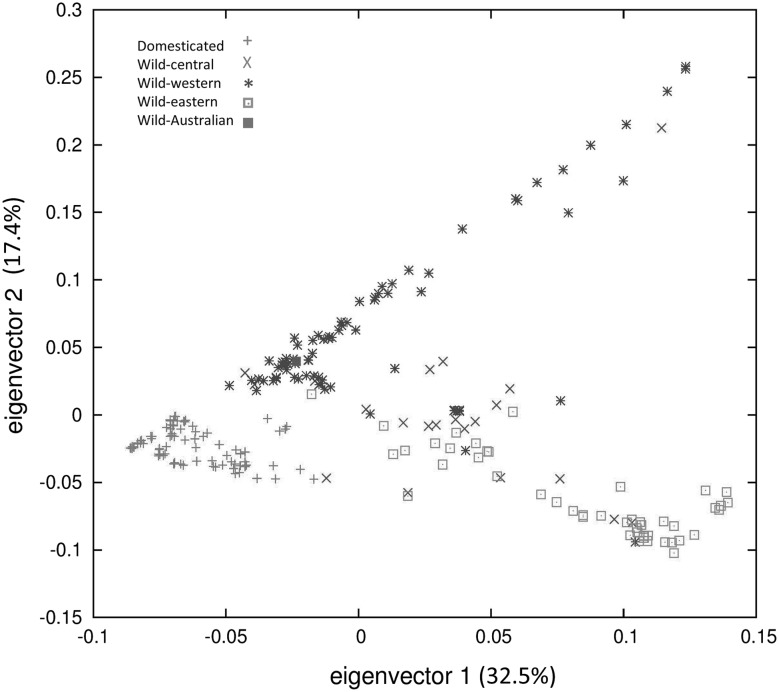
Principal component analysis (PCA) for 231 accessions of domesticated (Australian and European) and wild narrow-leafed lupin, labelled by their status

**Fig. 3:**
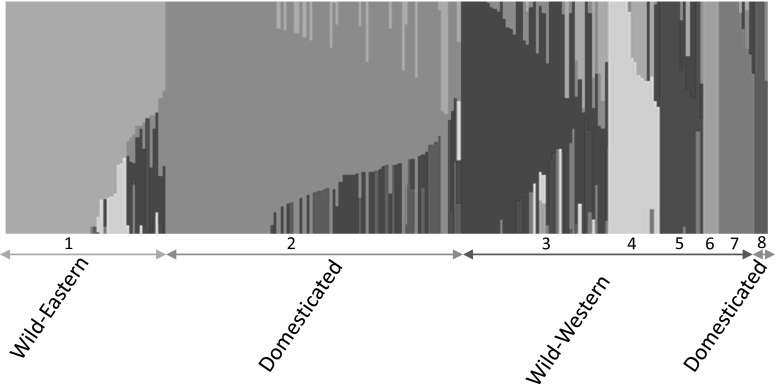
Population stratification among germplasm of wild and domesticated narrow-leafed lupin (*K* = 8) using fastSTRUCTURE. Colours denote population affiliation. Population 1 is the eastern wild group. Populations 2 and 8 are domesticated groups. The remaining five populations are western wild groups. For the assignment of accessions to each population and admixed group, see Online Resource 1

Principal component analysis (PCA) further supported the division of wild from domesticated accessions (Fig. 2). The first and second principal components explained 32.9% and 17.4% of the genetic diversity among all accessions, respectively. The first four principal components together accounted for 73% of observed variance. In order to probe further the relationships between accessions, a Bayesian clustering algorithm applied in fastSTRUCTURE program (Raj et al. 2014) was used. To reveal population structure, we investigated different numbers of populations from two to twelve (*K*2–12) (Online Resource 6). The internal algorithm in fastSTRUCTURE for multiple choices of *K* determined that population numbers *K* = 8 and *K* = 9 best explained the variation in the dataset. In order to determine which one of these two models (*K* = 8 or *K* = 9) better fit the data, we subjected both models to our phylogeny analysis (not presented). Again, as both models appeared plausible, the simpler model (*K* = 8) was selected (Fig. 3). We used a frequency threshold of > 0.7 to assign accessions to their corresponding populations. Where accessions had population affinity values below 0.7, they were categorised as Admixed. Structure grouping depicted eight populations: two populations for the domesticated accessions (Populations 2 and 8) and the remaining six populations for the wild accessions. Population 2 included most European and all Australian domesticated accessions, while population 8 contained three domesticated accessions from Belarus (Apba, Baga-18 and Vada). Overall, European domesticated accessions showed a higher proportion of admixture than Australian domesticated accessions, 9 out of 56 European domesticated accessions (16.1%) compared to 2 out of 29 Australian domesticated accessions (6.9%). Population 1 is a mainly eastern group with the exception of one accession from Portugal (P26423) and two accessions from Italy (P20716 and P26668). All other groups are western Mediterranean with only five exceptions from central Mediterranean (P25066, P25069, P25083, P25084 and P20720). For assignment of accessions to populations and admixed accessions, see Online Resource 1.

### Genome-wide pattern of LD decay

**Fig. 4:**
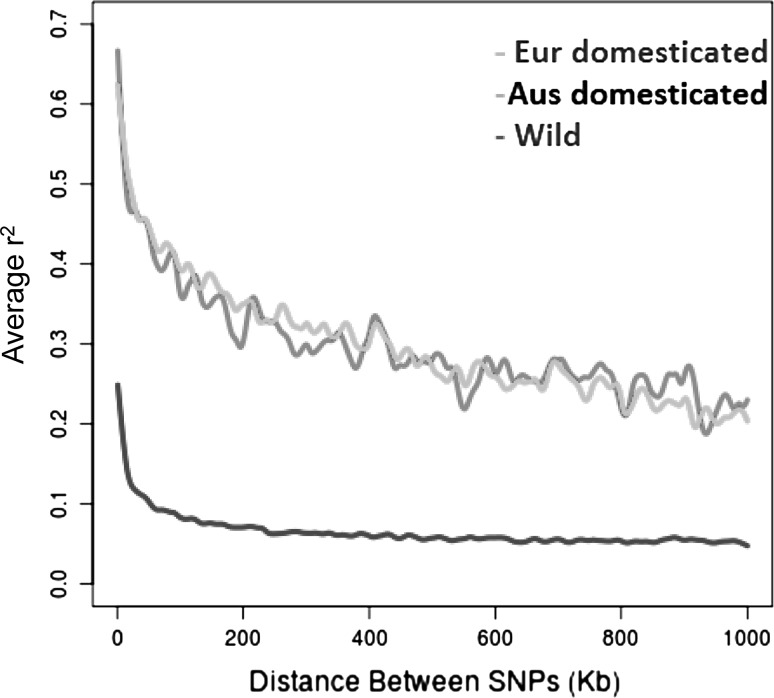
Comparison of genome-wide decay of linkage disequilibrium between wild and domesticated (Australian and European) accessions of narrow-leafed lupin

Differentiation of genetic diversity between these populations of wild and domesticated (sub-divided into Australian and European groups) narrow-leafed lupin was investigated further through analysis of decay of linkage disequilibrium (LD). The overall pattern of LD decay of European and Australian accessions was similar (*r*^2^ = 0.65 and 0.69 for European and Australian populations, respectively). However, LD was much lower in wild accessions (*r*^2^ = 0.27) compared to the domesticated. In addition, the LD decayed over a much shorter physical distance in the wild (19.01 Kb) compared to the domesticated populations (both European and Australian domesticated populations decayed at 77.45 Kb) (Fig. 4). The average nucleotide diversity (pi) per site showed that diversity was almost three times lower in domesticated population (0.097) compared to the wild population (0.271).

### Footprints of selection

**Fig. 5:**
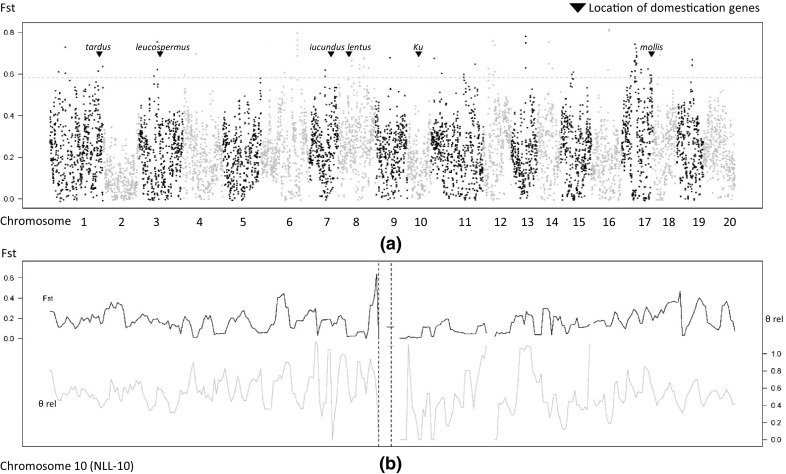
Fst-based genome-wide analysis of population differentiation within narrow-leafed lupin (*L. angustifolius*). **a** Fst estimated between wild and domesticated samples across the genome in non-overlapping windows of 1 Mb. Alternating black and grey sets of points correspond to the 20 pseudo-chromosomes of narrow-leafed lupin genome. Horizontal dashed line marks the .99 percentile of distribution of Fst estimated genome-wide. Inverted triangles denote expected location of domestication gene regions. **b** Fst (top) and relative polymorphism (domesticated/wild, bottom) estimated in overlapping windows of 200 Kb (50 Kb step) across pseudo-chromosome NLL-10. Vertical dashed lines denote the closest flanking markers to the candidate gene Ku. Some windows are missing due to the absence of SNP data

A selective sweep affecting only the domesticated narrow-leafed lupin would be expected to leave a typical high-divergence and low-polymorphism signal around the region of the selected gene. We focused here on the regions around domestication loci responsible for traits that were known *a priori* to have been selected during domestication: *Ku*, *lentus*, *tardus*, *mollis*, *iucundus* and *leucospermus* (Fig. 5a). The location of these genes is described in Hane et al. (2017). These six genomic regions encompassing domestication loci had levels of Fst indistinguishable from the rest of the genome (Fig. 5a), except perhaps for a weak signal around the *Ku* locus, which was associated with a decrease in the relative amount of polymorphism between domesticated and wild accessions (Fig. 5b). However, it should be emphasised that the more stringent BayeScan analysis based on FDR 0.01 and 0.05 showed no statistically significant signature of selection (Online Resource 7).

### Identifying SNP markers associated with domestication genes

**Fig. 6:**
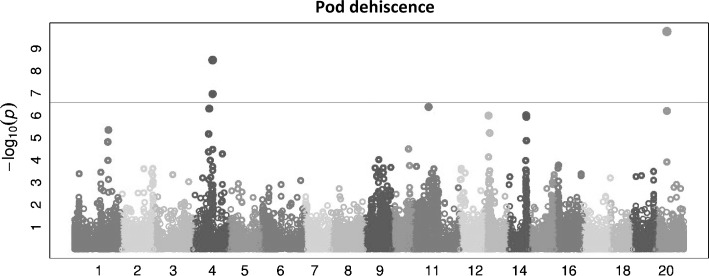
Manhattan plot of genome-wide association study (GWAS) using 38,948 SNPs markers for pod dehiscence on 231 wild and domesticated narrow-leafed lupin accessions. The x-axis represents physical distance (Kb) along the 20 narrow-leafed lupin chromosomes, NLL-01 to NLL-20. SNPs above the threshold line (green line; − log10(p) = 6) are significantly associated with pod dehiscence

Associations between markers and phenotypic traits (i.e. flowering time, flower colour, hard/soft seededness, alkaloid status, pod dehiscence, height at maturity and 100 seed weight) were examined. Strong associations were found between markers and flowering time, flower colour, alkaloid status and seed dehiscence. However, taking into account FDR-adjusted p values of these associations, only two SNPs related to pod dehiscence remained highly significant. One of these SNPs is located on position 8,766,720 bp of chromosome NLL-20 (DS_Lan_38166; FDR-adjusted p value of 6.42E−06; estimated allelic effect of −0.225), and the other is located on position 15,432,293 of chromosome NLL-04 (DS_Lan_07875; FDR-adjusted p value of 6.10E−05; estimated allelic effect of 0.184). A Manhattan plot of GWAS for pod dehiscence is presented in Fig. 6. For Manhattan plots of other traits, please see Online Resource 8. The genic composition of the whole regions within a window of 50 kb on each side of the markers associated with pod dehiscence trait was scanned as regions containing potential gene candidates. This revealed 8 and 7 genes on NLL-04 and NLL-20, respectively, around the SNPs associated with pod dehiscence. These genes and their Gene Ontology (GO) terms are listed in Online Resource 9. No standout candidate genes based on GO functional annotations were identified.

## Discussion

### Founder population of domesticated narrow-leafed lupin, admixture and genome-wide impact of domestication

We provide the first molecular evidence of a western Mediterranean origin of the domesticated gene pool of the narrow-leafed lupins, supporting earlier speculation based on morphological similarities (Gladstones 1998). A distinct separation of wild and domestic germplasm was supported by PCA and phylogeny analyses (Figs. 1 and 2). The incidence of four exceptions from the central population (P20711, P20726, P26107 and P26109) within the domesticated cluster could be explained by historic admixture as supported by fastSTRUCTURE analysis where these four accessions were identified as admixed (Fig. 3; Online Resource 1).

In the wild cluster, only one western Mediterranean accession (P28221) was placed in an eastern Mediterranean cluster and one eastern accession placed in a western cluster (P26446) (Fig. 1). This high level of congruity with geographic origin may reflect the low-level migration between the eastern and western Mediterranean, as shown by Mousavi-Derazmahalleh et al. (2018b). While narrow-leafed lupin is predominantly a self-pollinated species, different rates of natural cross-pollination (from 0 to 11.95%), in particular through honey bees, have been reported for samples from different geographical regions (Forbes et al. 1971). This indicates gene flow between wild and domesticated narrow-leafed lupin, which is due to either random cross-pollination or previously observed west/east migration patterns (Mousavi-Derazmahalleh et al. 2018b).

Consistent with the phylogeny result, PCA also clearly differentiated wild and domesticated samples. Distribution of wild accessions along the two different lines (Fig. 2) is due to the west/east division of wild samples which was discovered in our previous study (Mousavi-Derazmahalleh et al. 2018b). As with the phylogeny result (Fig. 1), wild accessions close to the domesticated accessions in the PCA plot (Fig. 2) are from the western Mediterranean, further supporting the western Mediterranean as the origin of domesticated narrow-leafed lupin. Interestingly, phylogeny and PCA results clearly show that domesticated samples from Australia and Europe are not genetically distinctive, indicating shared ancestry and/or interchange of breeding materials. The only exceptions of this are three domesticated Belarusian accessions that form their own fastSTRUCTURE population (*K*8; Fig. 3), which all have a shedding phenotype normally associated with wild plants (Online Resource 1). In the phylogenetic tree (Fig. 1), these three accessions are in the same branch with two central wild accessions (P26107 and P26109) and the root of the branch is connected to the wild eastern accessions (P28485 from Belarus). Six further Belarusian cultivated accessions were classified as admixed (Elena-9, G6, G12, LAKS-2, V5 and V7; Online Resource 1). Therefore, we deduce that a lot of crossing with wild material happened in the Belarusian breeding programme, which consequently caused their population structure to be different from other domesticated accessions. Unintended cross-pollination with wild populations can be ruled out because, like all narrow-leafed lupin breeding programmes, the Belarusian programme falls outside the distribution of wild narrow-leafed lupin across the Mediterranean Basin (Gladstones 1998).

Despite significant efforts over several decades to broaden genetic diversity in Australian narrow-leafed lupin cultivars using crosses with wild accessions (Berger et al. 2013), there was little evidence of admixture in Australian cultivars (Fig. 3; Online Resource 1). Only two Australian accessions (from a total of 29) were considered admixed: Belara and Yorrel (Online Resource 1). Both cultivars arose from crosses between cultivars and wild types from the western Mediterranean (Cowling 1999). This highlights the difficulty in effectively using genetic diversity in wild germplasm and the need for sophisticated crossing strategies (Berger et al. 2013; Cowling et al. 2009).

The genome-wide effect of domestication in narrow-leafed lupin as revealed by our study showed almost threefold reduction in genetic diversity from the wild plants (pi = 0.271) to domesticated narrow-leafed lupin (pi = 0.097) plants. Unfortunately, due to very recent domestication (< 100 years) and very high linkage disequilibrium in the domesticated population, it was not possible to estimate the extent of the bottleneck required for this reduction in genetic diversity. The existing demographic modelling approaches, such as implemented in dadi software (Gutenkunst et al. 2009), typically assume that segregating sites sampled across the genome are independent from each other. Due to high linkage disequilibrium in the domesticated accessions, the segregating sites are not independent, which was the likely cause of non-convergence of the analyses in our demographic modelling (not shown).

Decreased genetic diversity following domestication is generally accepted for most plants (Gepts 2014), for example rice (Li et al. 2011; Zhu et al. 2007), soybean (Hyten et al. 2006), common-bean (Sonnante et al. 1994) and maize (Vigouroux et al. 2005). However, contrasting results have been reported including a study of outcrossing species such as carrots (*Daucus carota*) (Iorizzo et al. 2013), apple (Gross et al. 2014) and tea (*Camellia taliensis*) (Zhao et al. 2014), where there was no significant reduction in genetic diversity during domestication. The narrow base of genetic diversity in domesticated narrow-leafed lupin compared to their wild type is the result of a severe founder effect that the crop experienced due to its short domestication history (Berger et al. 2012). Interestingly, a recent study demonstrated that the severe founder effect that narrow-leafed lupin plant experienced is not only limited to domestication of the crop, but also occurs in the eastern Mediterranean wild type (Mousavi-Derazmahalleh et al. 2018b). This observation was attributed to the migration of wild narrow-leafed lupin from western to eastern Mediterranean.

Congruent with our genetic diversity results, we observed a significant increase in LD across the whole genome from wild to domesticated germplasm. While LD was slightly higher in Australian domesticated narrow-leafed lupin than their European counterparts, their overall LD decay pattern was similar (Fig. 4). This observed small difference between these two populations is most probably due to the smaller population size of Australian accessions (29 accessions) in our study compared to the European accessions (56 accessions). In comparison, much lower LD was observed in the wild population (Fig. 4) which could be ascribed to phenomena such as their larger population size and higher levels of historic recombination.

### Evidence of selection

In general, Fst divergence and marker polymorphism were quite variable across the genome (Fig. 5a). We identified genomic regions with elevated Fst between wild and domesticated samples; however, these appeared not to be especially associated with regions known to harbour domestication genes. Indeed, no statistically supported signatures of selection based on the BayeScan method were found. There may be several reasons for this surprising lack of evidence for selective sweeps. One reason may be that narrow-leafed lupin has a very short and fragmented domestication history with severe population bottlenecks (Berger et al. 2012) resulting in very high levels of LD in domesticated populations (Fig. 4), which would tend to obscure signatures of selection. Another reason may be that European cultivars are not well described in terms of their pedigrees or domestication traits in contrast to Australian cultivars (Cowling 1999). There may be multiple sources of domestication genes in European cultivars, and some European cultivars may not be fully domesticated. For example, fully indehiscent seeds (controlled by *lentus* and *tardus* loci) are desirable, but not essential in northern European spring-sown cultivars because they experience cool, damp autumn harvest conditions in contrast to autumn-sown cultivars in Australia that experience hot, dry spring harvest conditions such that both indehiscence loci are essential. Finally, some domestication traits may be under opposite selective pressures in both wild and domesticated populations. For example, the hot, dry lupin-growing areas of Western Australia favour cultivars with early phenology to allow crops to mature before the onset of summer; in contrast, the cooler, wetter growing regions of eastern Australia favour later phenology cultivars to maximise grain yield (Berger et al. 2012). This adaptation is achieved largely by two allelic forms of the *Ku* locus, which has been identified as an *FT* homologue (Nelson et al. 2017). There is also emerging evidence that the *Ku* locus mediates phenological adaptation in wild populations (Mousavi-Derazmahalleh et al. 2018b; Taylor et al. 2018). This complexity may explain why a short spike in Fst and small reduction in polymorphism was observed around the *Ku* locus in this study (Fig. 5b) despite its known importance in phenological adaptation.

Lack of clear signatures of selection around known domestication genes has also been reported in other systems. For example, Hufford et al. (2012) found the strongest selection in maize to have occurred in genes other than well-established domestication genes, implying that hundreds of genes with a variety of biological functions have been targets of selection although their phenotypic effect may be still unknown. Other studies in maize identified non-coding regions as playing important regulatory roles in crop domestication (Jiao et al. 2012; Yu et al. 2012).

There are several ways in which these challenges may be addressed in future selective sweep analysis of narrow-leafed lupin. These include increasing population sample size (from 231 used in this study), increasing genotyping density (from 1 SNP per 13 kb in this study) using whole genome resequencing, better characterising the domestication status of European cultivars and by categorising both wild and domesticated accessions according to their allelic status at the *Ku* locus as recently started by Taylor et al. (2018).

### Genome-wide association study

Interestingly, the GWAS approach was more successful than Fst analysis in finding significant regions of the genome associated with domestication traits. We identified two regions of the genome significantly associated with pod indehiscence on chromosomes NLL-04 and NLL-20 (Fig. 6). Based on functional annotation information available for narrow-leafed lupin, there were no evident gene candidates for pod dehiscence in those regions (Online Resource 9). This may be due to our lack of understanding of the underlying biology of pod indehiscence, incomplete assembly of the Tanjil reference genome (an estimate half of the genome is represented in pseudo-chromosomes; Hane et al. 2017) or incomplete gene annotation (8 out of 15 genes identified were unannotated; Online Resource 9). An improved genome assembly is clearly a priority for narrow-leafed lupin research including population genetics. Nevertheless, these genomic regions provide starting points for future genome resequencing studies in larger sets of accessions to identify genes associated with domestication.

### Summary conclusion and future direction

For the first time, this study confirmed that the western Mediterranean provided the founder population of domesticated narrow-leafed lupin. This will contribute to the efficient collecting of genetic resources for future breeding plans. Furthermore, since the methods applied in this study for revealing the footprints of selection in the genome did not satisfy expectations, it would be interesting to repeat this analysis with an expanded set of well-characterised germplasm and higher-resolution genotyping by whole-genome resequencing.

#### Author contribution statement

MMD and MNN conceived the design of this study with input on analytical approaches by DF, BN, DE, PEB, WE and JKH. BN and DF conducted the selective sweep and genome-wide divergence analyses and its writing. MMD, JKH and PEB identified SNP positions related to narrow-leafed reference genome. MMD conducted all other analyses. MMD and MNN led the manuscript preparation. All authors have read and approved the final manuscript.

## Electronic supplementary material

Below is the link to the electronic supplementary material.

Online Resource 1 Phenotypic data and country of origin information for 231 wild and domesticated narrow-leafed lupin accessions and three technical replicates from the Australian Lupin Collection, South Perth, Australia (XLSX 29 kb)

Online Resource 2 The allelic profile per accession for 45,230 SNP markers, applied on 231 wild and domesticated accessions of narrow-leafed lupin (XLSX 37478 kb)

Online Resource 3 The allelic profile per accession for 11,690 SNP markers, applied on 231 wild and domesticated accessions of narrow-leafed lupin (XLSX 9868 kb)

Online Resource 4 Unrooted radial phylogenetic tree of wild and domesticated narrow-leafed lupin constructed from 11,690 SNPs, using MrBayes v3.2.2. (PDF 15 kb)

Online Resource 5 NeighborNet network for the 231 wild and domesticated accessions of narrow-leafed (PDF 41 kb)

Online Resource 6 Population stratification among 231 wild and domesticated accessions of narrow-leafed lupin using fastSTRUCTURE for K = 2–12. Each colour denotes a population affiliation. Note that colours are not equivalent between panels (PPTX 138 kb)

Online Resource 7 BayeScan plot of Fst compared with Log 10 (q value) (PDF 8 kb)

Online Resource 8 Manhattan plots of genome-wide association study (GWAS) using 38,948 SNPs markers for alkaloid status, flowering time and flower colour on 231 wild and domesticated narrow-leafed lupin accessions. The X-axis represents physical distance (Kb) along the 20 narrow-leafed lupin chromosomes, NLL-01 to NLL-20. SNPs above the threshold line (green line; -log10(p) = 6) are significantly associated with related traits (PPTX 287 kb)

Online Resource 9 Gene content of the genomic regions close to SNPs associated with pod dehiscence, located on chromosome NLL-20 and NLL-04, respectively (XLSX 10 kb)
