# Supplementary figures and images for: The western Mediterranean region provided the founder population of domesticated narrow-leafed lupin
Source: Theor Appl Genet. 2018 Sep 17;131(12):2543–54. doi: 10.1007/s00122-018-3171-x (PMC6244526; doi:10.1007/s00122-018-3171-x)

Domesticated

Wild-central

## Wild-western

## Wild-eastern

## Wild-Australian

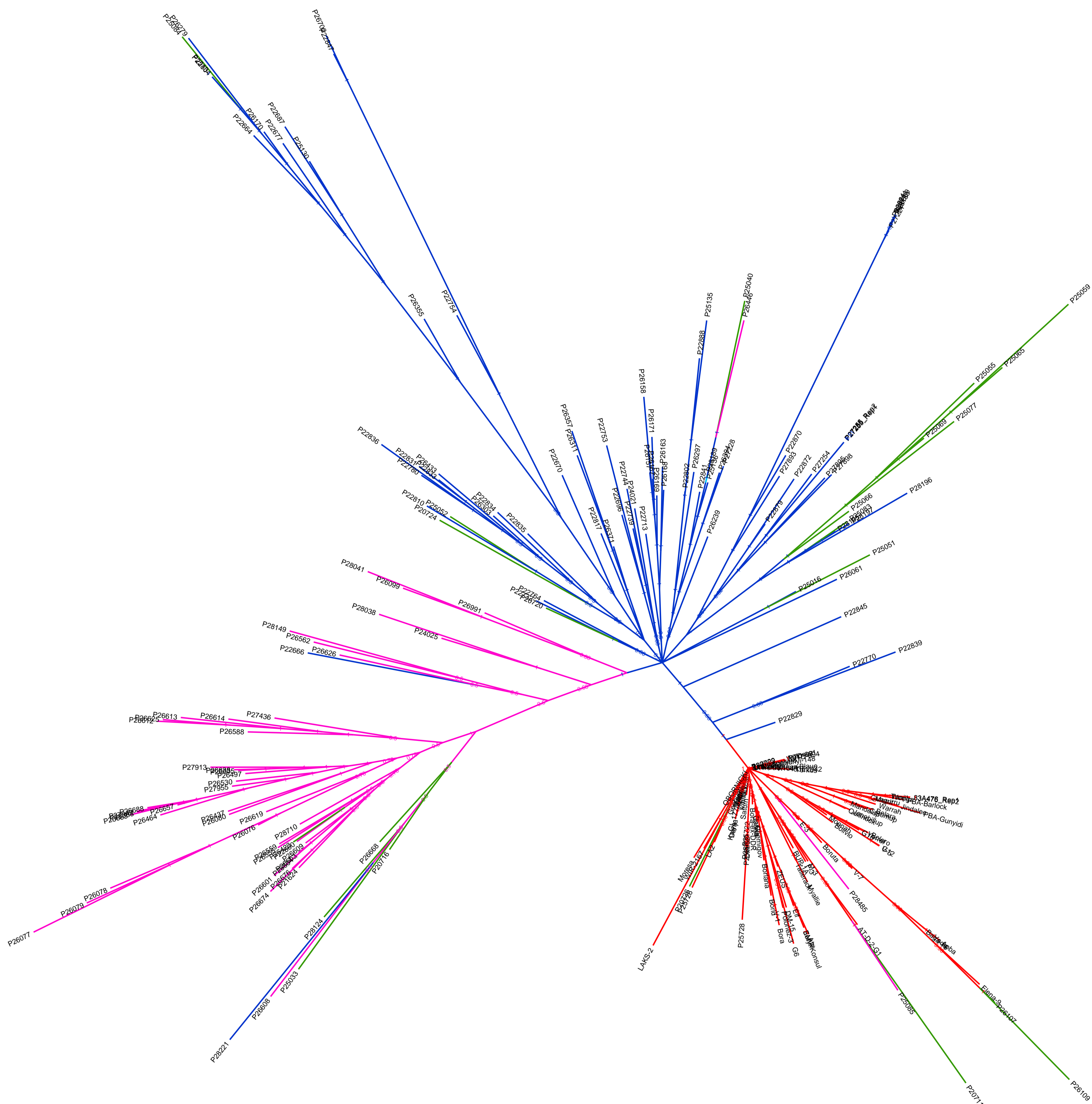

Supplement: Supplementary file 4 — Online Resource 4 Unrooted radial phylogenetic tree of wild and domesticated narrow-leafed lupin constructed from 11,690 SNPs, using MrBayes v3.2.2. (PDF 15 kb) [file 122_2018_3171_MOESM4_ESM.pdf]

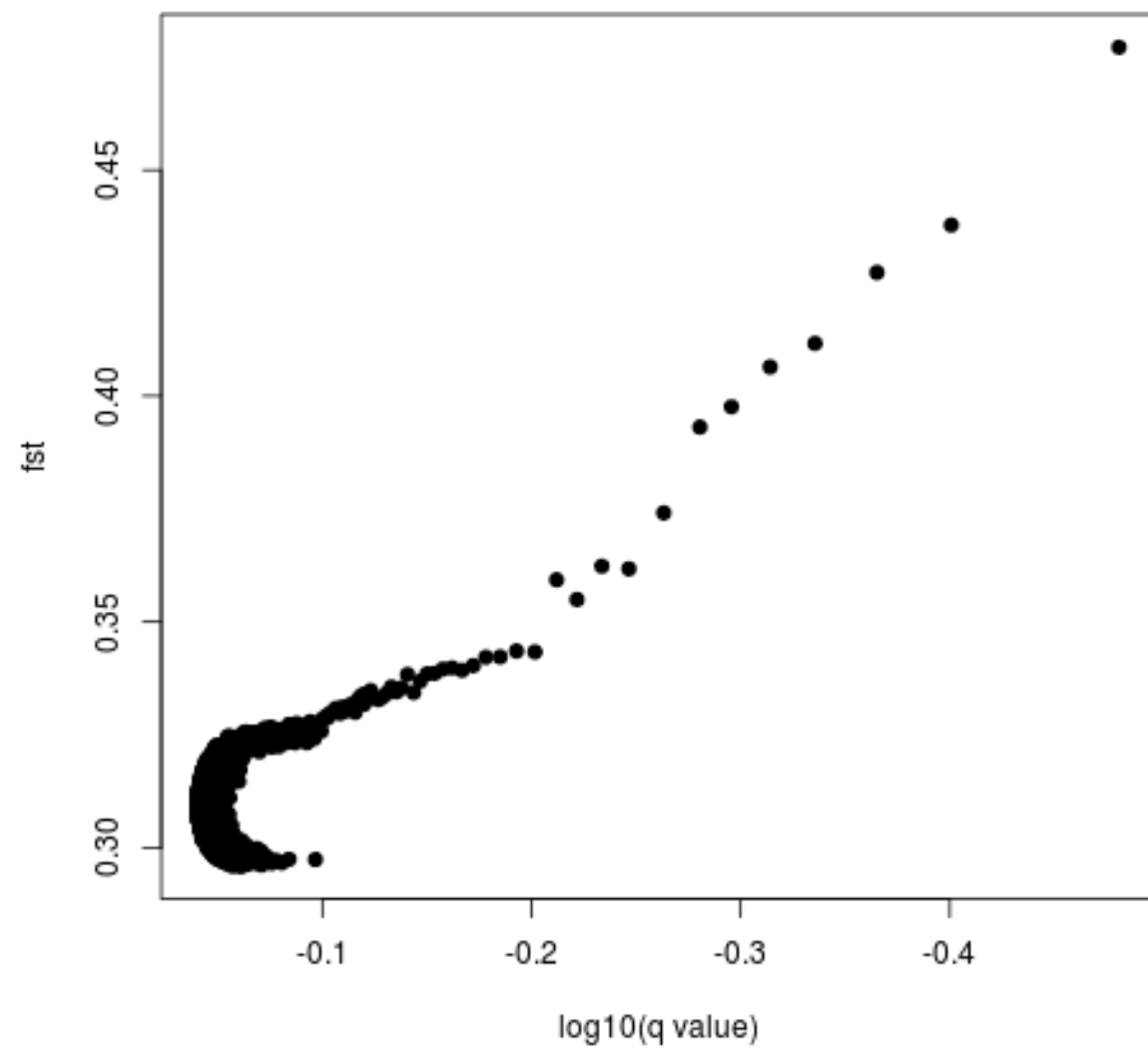

Supplement: Supplementary file 7 — Online Resource 7 BayeScan plot of Fst compared with Log 10 (q value) (PDF 8 kb) [file 122_2018_3171_MOESM7_ESM.pdf]
