# Supplementary material for: The western Mediterranean region provided the founder population of domesticated narrow-leafed lupin
Source: Theor Appl Genet. 2018 Sep 17;131(12):2543–54. doi: 10.1007/s00122-018-3171-x (PMC6244526; doi:10.1007/s00122-018-3171-x)
Supplement: Supplementary file 6 — Online Resource 6 Population stratification among 231 wild and domesticated accessions of narrow-leafed lupin using fastSTRUCTURE for K = 2–12. Each colour denotes a population affiliation. Note that colours are not equivalent between panels (PPTX 138 kb) [file 122_2018_3171_MOESM6_ESM.pptx]

## Slide 1
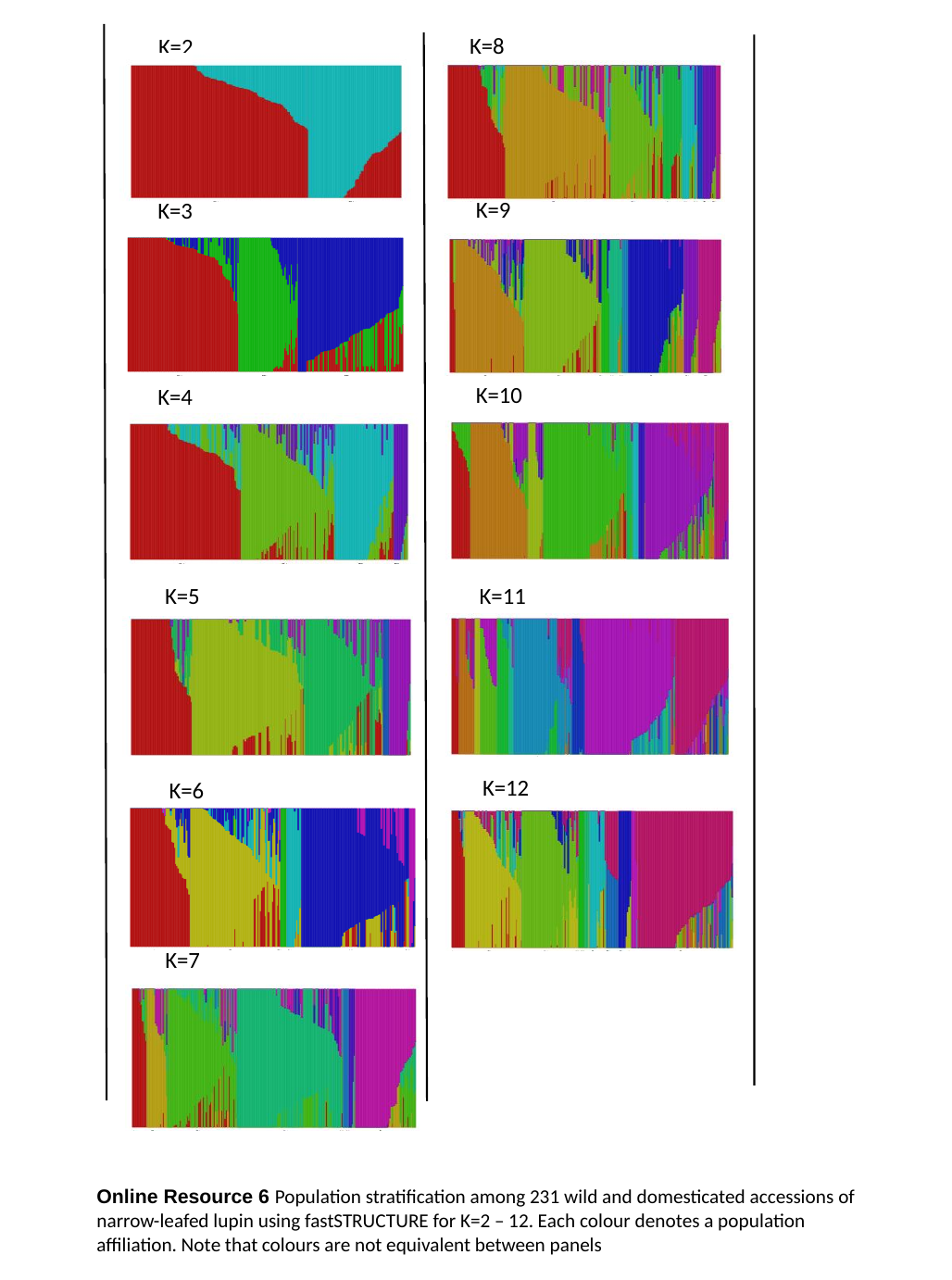

K=8
K=2
K=9
K=3
K=10
K=4
K=11
K=5
K=12
K=6
K=7
Online Resource 6 Population stratification among 231 wild and domesticated accessions of narrow-leafed lupin using fastSTRUCTURE for K=2 – 12. Each colour denotes a population affiliation. Note that colours are not equivalent between panels
