# Supplementary material for: The western Mediterranean region provided the founder population of domesticated narrow-leafed lupin
Source: Theor Appl Genet. 2018 Sep 17;131(12):2543–54. doi: 10.1007/s00122-018-3171-x (PMC6244526; doi:10.1007/s00122-018-3171-x)
Supplement: Supplementary file 8 — Online Resource 8 Manhattan plots of genome-wide association study (GWAS) using 38,948 SNPs markers for alkaloid status, flowering time and flower colour on 231 wild and domesticated narrow-leafed lupin accessions. The X-axis represents physical distance (Kb) along the 20 narrow-leafed lupin chromosomes, NLL-01 to NLL-20. SNPs above the threshold line (green line; -log10(p) = 6) are significantly associated with related traits (PPTX 287 kb) [file 122_2018_3171_MOESM8_ESM.pptx]

## Slide 1
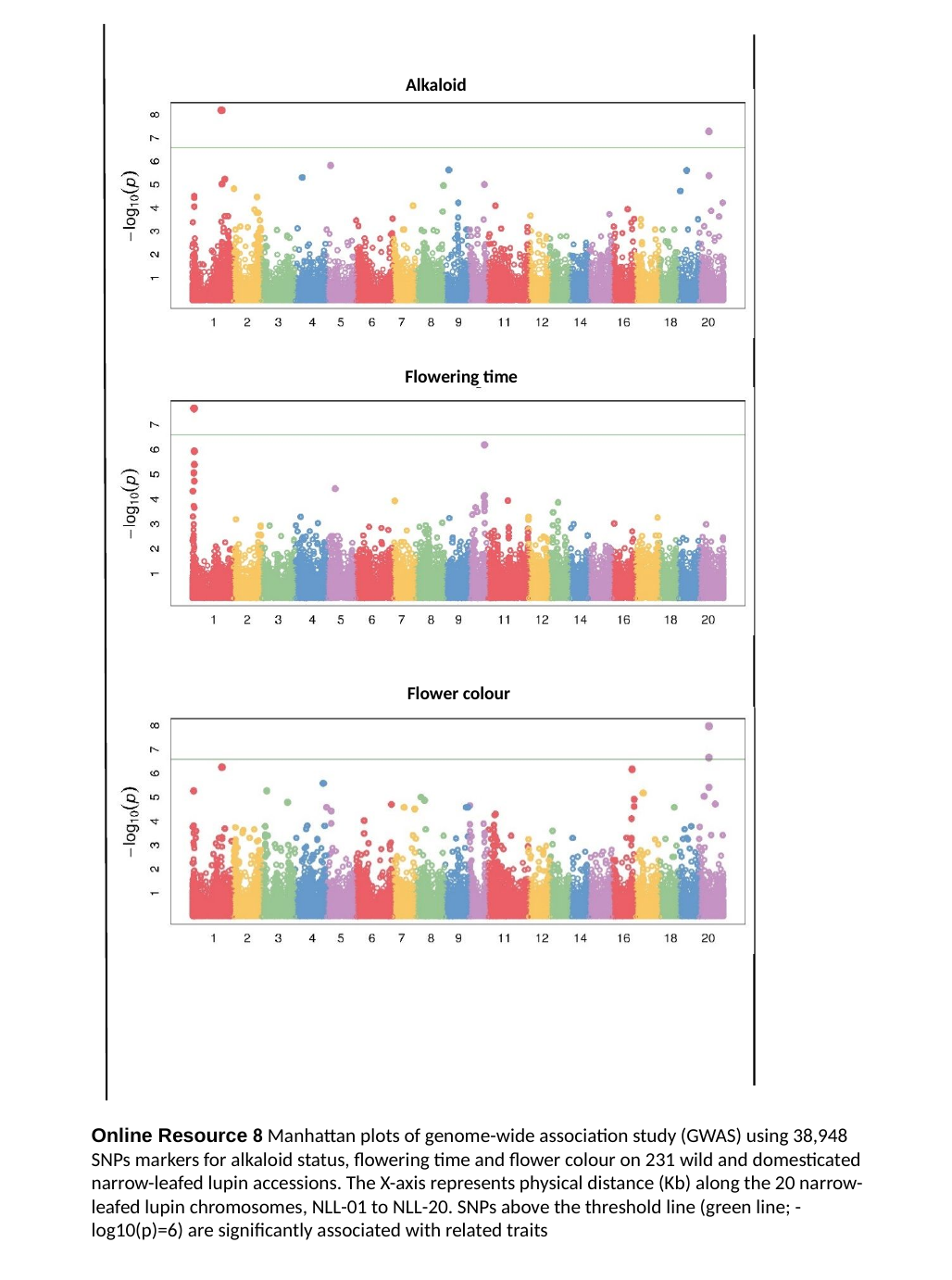

Alkaloid
Flowering time
Flower colour
Online Resource 8 Manhattan plots of genome-wide association study (GWAS) using 38,948 SNPs markers for alkaloid status, flowering time and flower colour on 231 wild and domesticated narrow-leafed lupin accessions. The X-axis represents physical distance (Kb) along the 20 narrow-leafed lupin chromosomes, NLL-01 to NLL-20. SNPs above the threshold line (green line; -log10(p)=6) are significantly associated with related traits
