# Supplementary material for: The western Mediterranean region provided the founder population of domesticated narrow-leafed lupin
Source: Theor Appl Genet. 2018 Sep 17;131(12):2543–54. doi: 10.1007/s00122-018-3171-x (PMC6244526; doi:10.1007/s00122-018-3171-x)
Supplement: Supplementary file 5 — Online Resource 5 NeighborNet network for the 231 wild and domesticated accessions of narrow-leafed (PDF 41 kb) [file 122_2018_3171_MOESM5_ESM.pdf]

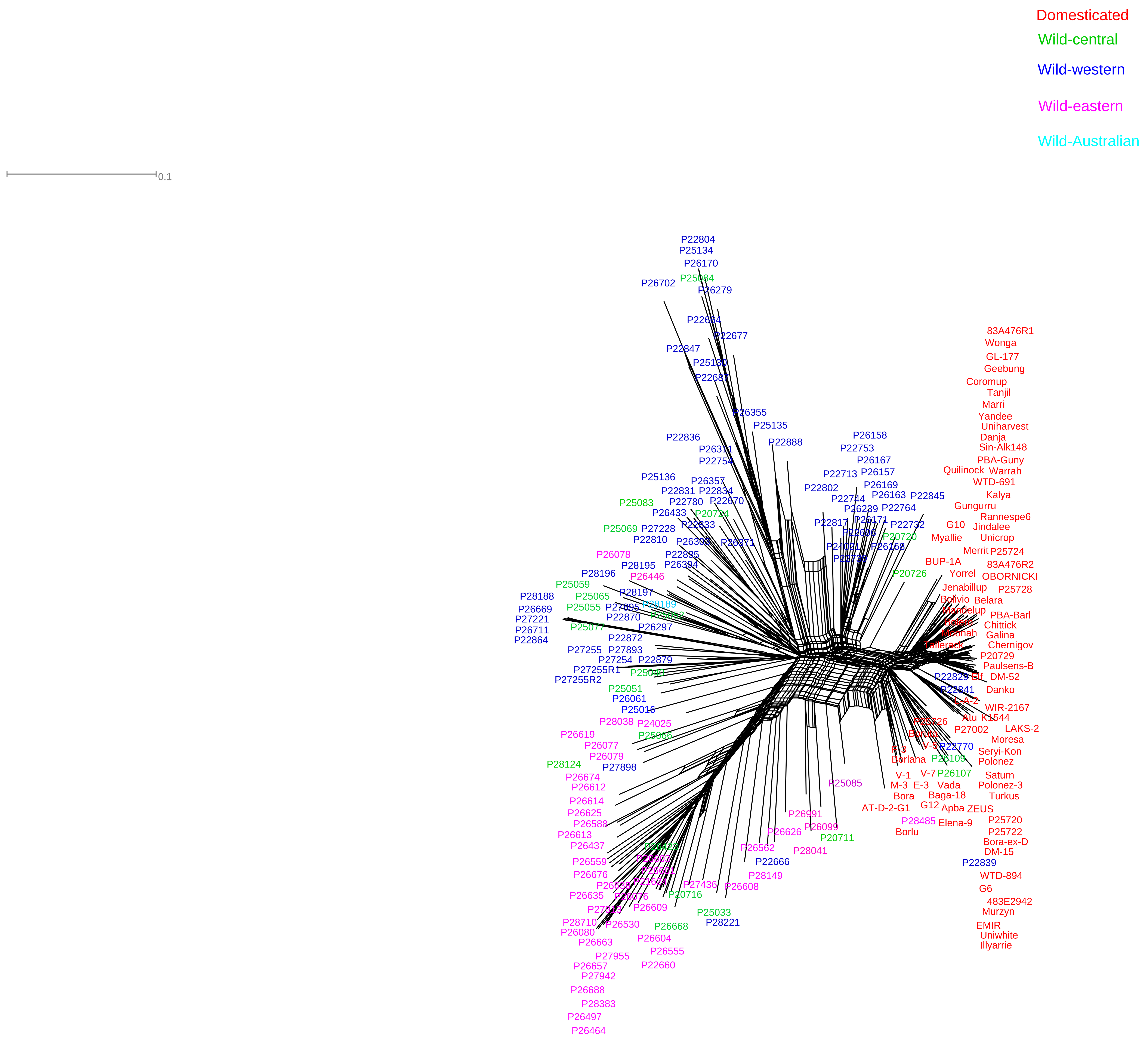

Domesticated
Wild-central
Wild-western
Wild-eastern
Wild-Australian
0.1
P22804
P25134
P26170
P26702
P25084
P26279
P22677
P22847
P26355
P25135
P22836
P22888
P26158
P22753
P26167
P22713
P26157
P22802
P26169
P26163
P22845
P22744
P22764
P26239
P22817
P22732
P25136
P26357
P22831
P22834
P25083
P22780
P22670
P26433
P25069
P27228
P22810
P22833
P20726
P26078
P28195
P26394
P28196
P26446
P25059
P28188
P25065
P28197
P26669
P25055
P27221
P22870
P26711
P25077
P26297
P22864
P22872
P27255
P27893
P27254
P22879
P27255R1
P27255R2
P25051
P26061
P25016
P28038
P24025
P26619
P26077
P26079
P28124
P27898
P26674
P26612
P26614
P26625
P26588
P26613
P26437
P26559
P26676
P26635
P26609
P28710
P26530
P26080
P26663
P26668
P26604
P25033
P28221
P26555
P27955
P22660
P26657
P27942
P26688
P28383
P26497
P26464
P20716
P27436
P26608
P28149
P22666
P26562
P28041
P26626
P26991
P26099
P20711
P25085
P28485
Borlu
AT-D-2-G1
83A476R1
Wonga
GL-177
Geebung
Coromup
Tanjil
Marri
Yandee
Uniharvest
Danja
Sin-Alk148
PBA-Guny
Quilinock
Warrah
WTD-691
Kalya
Gungurru
Rannespe6
G10
Jindalee
Myallie
Unicrop
Merrit
P25724
BUP-1A
83A476R2
Yorrel
OBORNICKI
Jenabillup
P25728
Belara
PBA-Barl
Chittick
Galina
Chernigov
P20729
Paulsens-B
DM-52
P22841
Danko
WIR-2167
K1544
P27002
LAKS-2
P22770
Moresa
Seryi-Kon
Polonez
Borlana
P26107
V-1
V-7
Saturn
M-3
E-3
Vada
Polonez-3
Bora
Baga-18
Turkus
G12
Apba
ZEUS
Elena-9
P25720
P25722
Bora-ex-D
DM-15
P22839
WTD-894
G6
483E2942
Murzyn
EMIR
Uniwhite
Illyarrie
